# Acute haematogenous community-acquired methicillin-resistant *Staphylococcus aureus* osteomyelitis in an adult: Case report and review of literature

**DOI:** 10.1186/1471-2334-12-270

**Published:** 2012-10-25

**Authors:** Amreeta Dhanoa, Vivek Ajit Singh, Azura Mansor, Mohd Yasim Yusof, King-Ting Lim, Kwai-Lin Thong

**Affiliations:** 1 Jeffrey Cheah School of Medicine and Health Sciences, Monash University Sunway Campus, 46150, Bandar Sunway, Malaysia; 2 Department of Orthopaedic Surgery, Faculty of Medicine, University of Malaya, 50603, Kuala Lumpur, Malaysia; 3 Department of Medical Microbiology, Faculty of Medicine, University of Malaya, 50603, Kuala Lumpur, Malaysia; 4 Institute of Biological Science, Faculty of Science, University of Malaya, 50603, Kuala Lumpur, Malaysia

**Keywords:** Community-acquired MRSA, Osteomyelitis, Adult, Pyomyositis, Septic arthritis, Bacteraemia, PVL, Inflammatory markers, Osteosarcoma mimicker

## Abstract

**Background:**

Methicillin-resistant *Staphylococcus aureus* (MRSA) has of late emerged as a cause of community-acquired infections among immunocompetent adults without risk factors. Skin and soft tissue infections represent the majority of community-acquired methicillin-resistant *Staphylococcus aureus* (CA-MRSA) clinical presentations, whilst invasive and life-threatening illness like necrotizing pneumonia, necrotizing fasciitis, pyomyositis, osteomyelitis and sepsis syndrome are less common. Although more widely described in the pediatric age group, the occurrence of CA-MRSA osteomyelitis in adults is an uncommonly reported entity.

**Case presentation:**

We describe an invasive CA-MRSA infection in a 28 year-old previously healthy male, manifesting with bacteraemia, osteomyelitis of femur, pyomyositis and septic arthritis of the knee. Initially a preliminary diagnosis of osteosarcoma was suggested by imaging studies and patient underwent a bone biopsy. MRSA was subsequently isolated from blood cultures taken on day of admission, bone, tissue and pus cultures. Incision and drainage of abscess was performed and patient was treated with vancomycin, with fusidic acid added later. It took 6 months for the inflammatory markers to normalize, warranting 6-months of anti-MRSA therapy. Patient was a fervent deer hunter and we speculate that he acquired this infection from extensive direct contact with deer.

Molecular characterization of this isolate showed that it belonged to multilocus sequence type (MLST) ST30 and exhibited the staphylococcal chromosome cassette *mec* (SCC*mec*) type IV, staphylococcus protein A (*spa*) type t019, accessory gene regulator (*agr*) type III and *dru* type dt10m. This strain harbored Panton-Valentine leukocidin (*pvl*) genes together with 3 other virulent genes; *sei* (enterotoxin), *hlg* (hemolysin) and *fnbA* (fibronectin binding protein).

**Conclusion:**

This case study alerts physicians that beyond the most commonly encountered skin and soft tissue infections, *pvl* positive CA-MRSA can lead to invasive life-threatening disease especially in an immunocompetent adult. Heightened alertness is needed for osteomyelitis of long bones in adults, as it is not uncommon for this disease to mimic primary bone malignancy. Cure is achievable with early appropriate antibiotics guided by inflammatory markers.

## Background

Although traditionally regarded a healthcare-associated pathogen, methicillin-resistant *Staphylococcus aureus* has now emerged as an important cause of infections in the community, with little or no links to healthcare setting [1-3]. Community-acquired methicillin-resistant *S. aureus* strains (CA-MRSA) carry the smaller staphylococcal chromosome cassette *mec* (SCC*mec*) types IV or V whereas the larger SCC*mec* types I, II and III are present in MRSA conventionally associated with infections in healthcare facilities [1]. The virulence of CA-MRSA may in part be related to its ability to produce toxins, such as Panton-Valentine leukocidin (PVL) which has an unique ability to kill leukocytes [2], resulting in bacterial evasion of bactericidal function of leukocytes [4]. Although initially associated with skin infections, PVL has recently been shown to play an essential role in the pathogenesis of invasive infections such as necrotizing pneumonia and osteomyelitis [1,2].

Overall, skin and soft-tissue infections represent about 77% to 90% of CA-MRSA infections and these are predominantly abscesses or cellulitis [2,3]. Although reported much less frequently, of greater concern are reports of invasive infections with CA-MRSA manifesting as bacteraemia, osteomyelitis, pneumonia and septic arthritis [3,4]. Osteomyelitis alone accounts for only 1% of all CA-MRSA infections [2,3] and has been widely described in the pediatric age group [5]. On the contrary, the occurrence of CA-MRSA osteomyelitis in adults is an uncommonly reported entity. Herein, we report an invasive CA-MRSA infection in a previously healthy male presenting with bacteraemia, osteomyelitis of femur, pyomyositis and septic arthritis. He is a fervent deer hunter and presumably acquired this infection from extensive contact with deer after a hunting venture. This strain of CA-MRSA harbored both SCC*mec* type IV and *pvl* genes together with 3 other virulent genes; *sei* (enterotoxin), *hlg* (hemolysin) and *fnb*A (fibronectin binding protein). We also reviewed the literature for previously reported cases of osteomyelitis caused by CA-MRSA in adults.

## Case presentation

### Clinical presentation, investigation and management

A 28 year-old-man presented with progressive pain and swelling on the left thigh for 2 weeks prior to admission associated with intermittent fever. This pain started off as a deep boring pain associated with rest pain and night pain. It progressively got worst till he was unable to bear weight on the affected limb and was bound to a wheelchair. In addition to that, he also experienced constitutional symptoms such as loss of appetite and loss of weight. He denied history of cough, night sweats, haemoptysis or recent contact with tuberculosis patients. There were no recent skin or soft tissue lesions in him or any of his household contacts. No exposure to healthcare settings or any hospital interventions were elicited within the past one year. There was no previous medical history and he had not received antibiotic treatment in the past 3 months.

Our patient is a civil servant who worked temporarily in Sabah, a state in East Malaysia. He is an avid deer hunter and 2 weeks prior to his clinical presentation he went deer hunting with a hunting partner and caught 3 deer from a forest in Tawau, a small town located at the south-east coast of Sabah. There was extensive direct contact with the deer carcass as he slaughtered the deer with a meat saw, skinned it, cut the deer meat into pieces and cleaned it up. They then cooked the deer meat and consumed it. Neither he nor his hunting partner had any food-poisoning symptoms after eating the meat. A few days after this hunting venture, the patient flew back to his family-home located about 1800 km from Tawau and was brought to the hospital by his family members as he was experiencing severe pain on his left thigh. According to our patient, his hunting partner also experienced fever and lower leg pain at about the same time as him. Unfortunately, we could not obtain any further history regarding his hunting partner as our patient lost contact with him once he left Tawau.

Apart from deer, he was not exposed to any other animals including companion animals. He is a regular smoker; however he does not use intravenous drugs and had no history of acupuncture, trauma, recent travel abroad or involvement in recreational activities like river rafting, diving or contact sports. He is single and denies homosexual behavior.

His vital signs on admission revealed a temperature of 38.7°C, heart rate of 90 beats/min and blood pressure of 120/78 mmHg. His left thigh was warm and tender with a deep ill-defined swelling measuring 20 cm by 8 cm. There were no discharging sinuses or other skin or soft tissue lesions noted. His left knee movement was restricted due to pain; however there was no swelling or tenderness of the knee joint. There was no inguinal lymphadenopathy and neurological and vascular testing was unremarkable. His lungs were clear both clinically and radiologically and examination of other systems was unremarkable.

**Figure 1:**
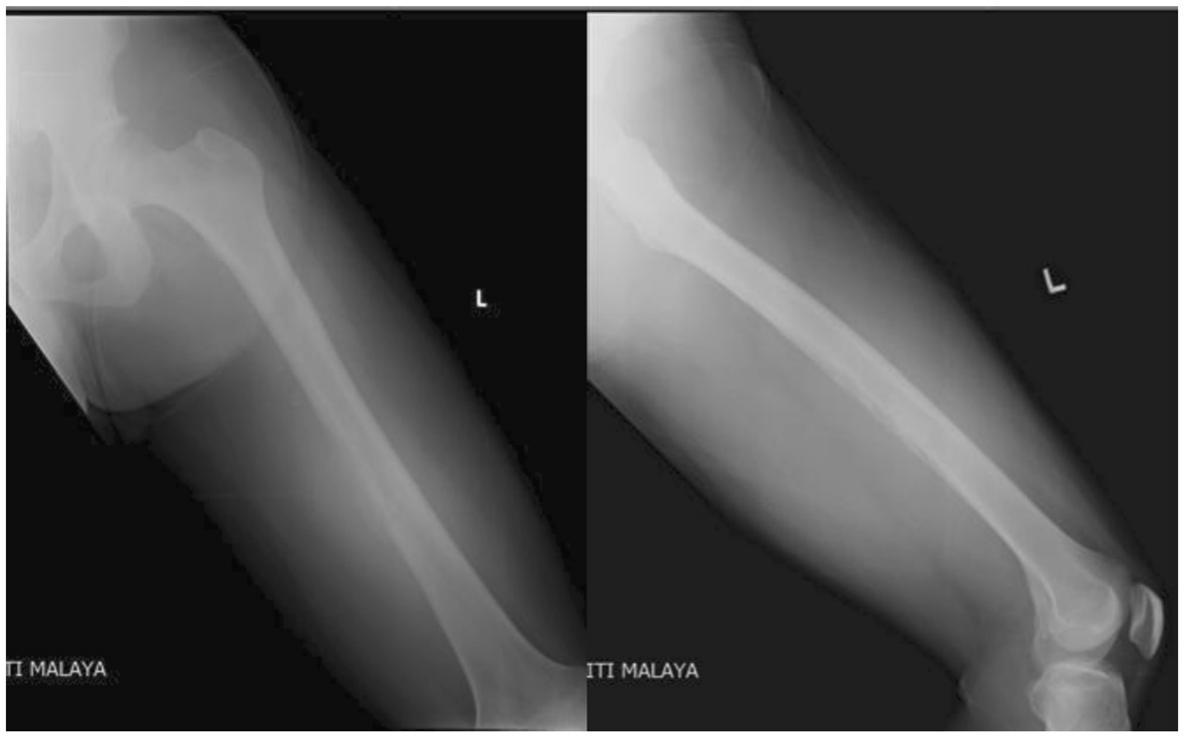
Plain radiograph shows destruction of the posterior medial cortex of the mid-shaft of left femur with periosteal reaction associated with a soft tissue shadow.

**Figure 2:**
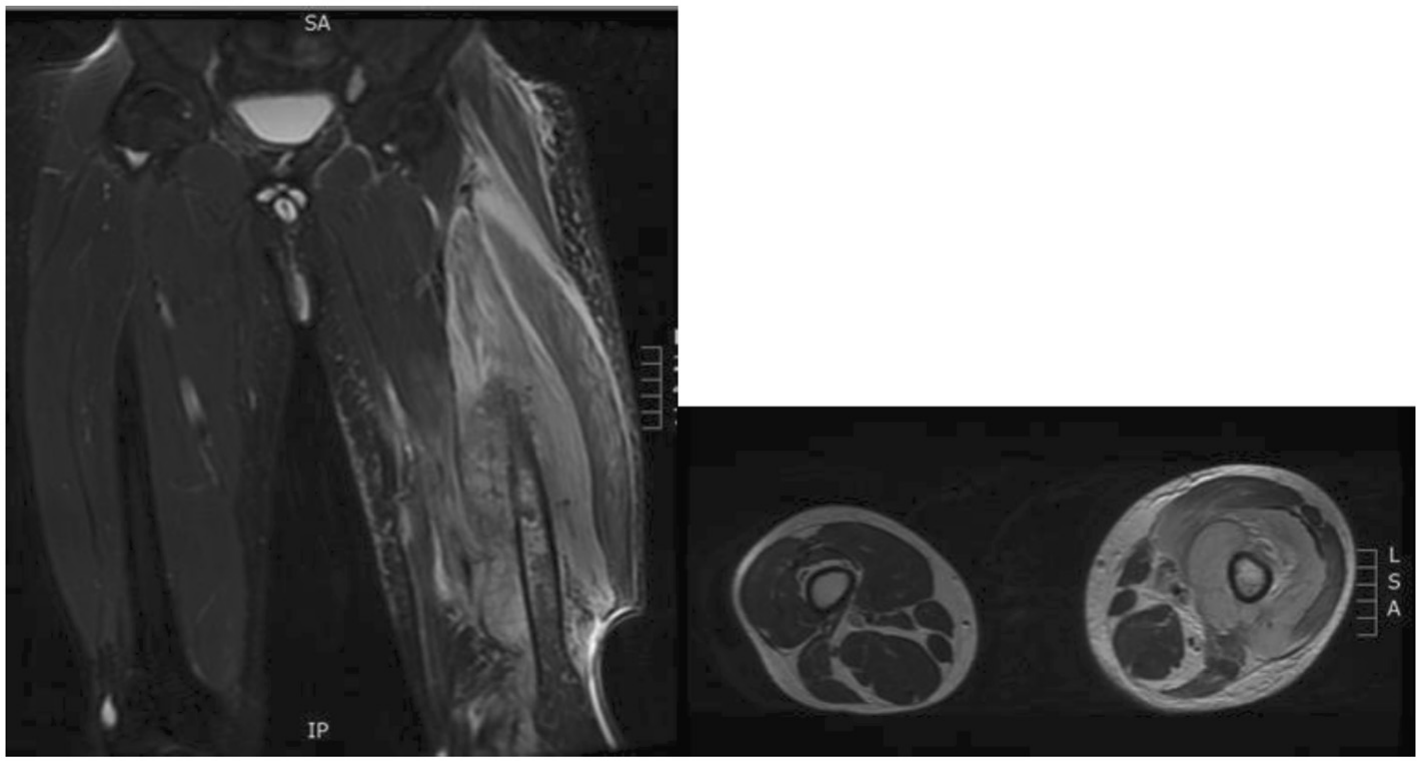
**Shows MRI sagittal and axial views of the both the femurs.** There are signal changes in the diaphyseal region of the left femur with circumferential soft tissue involvement.

**Figure 3:**
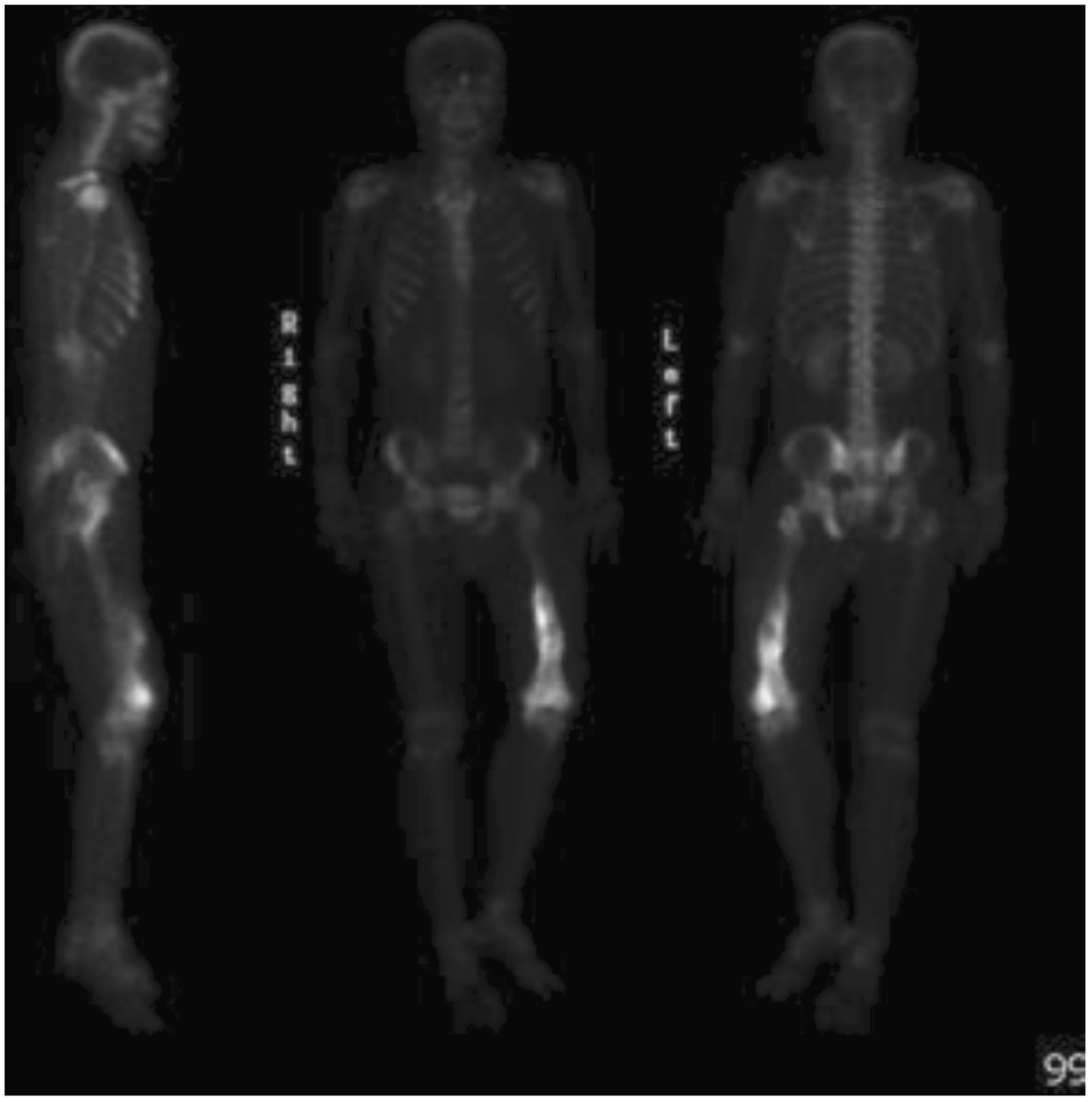
Bone scan showing isolated uptake of the left mid and distal femur.

Plain anteroposterior radiography revealed an osteolytic lesion with erosion of cortex and periosteal reaction involving the mid-shaft of the left femur (Figure 1). A magnetic resonance imaging done (MRI) showed involvement of the mid-shaft of left femur with surrounding circumferential soft tissue involvement (Figure 2). The technetium-99 m bone scan showed isolated uptake of the left mid and distal femur (Figure 3). A provisional diagnosis of osteosarcoma was made based on the radiological findings.

Initial blood investigations revealed leukocytosis (17.7 × 10^9^/L) with 86% neutrophils, normal haemoglobin and platelets. The C-reactive protein (CRP) and erythrocyte sedimentation rate (ESR) were both markedly elevated at 45.6 mg/dL and 140 mm/hour respectively. All other blood investigations were normal. Blood cultures were taken on day of admission and we proceeded to a needle biopsy. Three days later, both blood and bone biopsy cultures grew MRSA.

**Figure 4:**
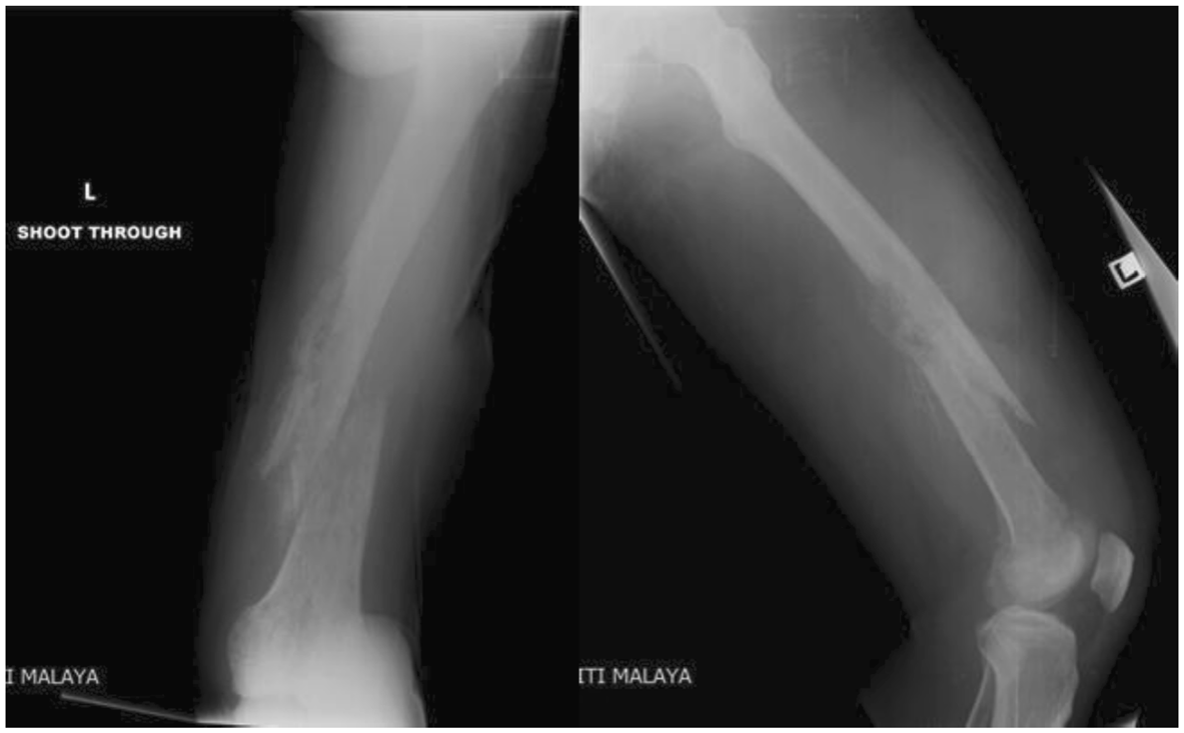
Radiograph demonstrating pathological fracture of the distal third of the left femur with shortening.

Patient was immediately commenced on intravenous vancomycin 1 gram bd, based on antibiotic sensitivity results. An incision and drainage was performed for the left thigh abscess and the cultures of pus also yielded MRSA. Management of the patient was complicated by a pathological fracture of the distal third of left femur sustained during an incision and drainage when a manipulation under anaesthesia was performed at the same time for the stiff knee (Figure 4). The fracture was managed with skin traction. Meanwhile, histopathological examination of the bone biopsy revealed acute on chronic suppurative inflammation with no evidence of granulomas or neoplastic cells. Ziehl- Neelsen stain did not reveal acid-fast bacilli and fungal stains were negative. A transthoracic echocardiogram done showed no evidence of valve vegetation, excluding the possibility of infective endocarditis. Ultrasound of abdomen did not reveal abscesses or other abnormalities. Placement of a peripherally inserted central catheter (PICC) line was done for long term antibiotics administration. Hepatitis B and HIV screening were performed and both were negative.

One month after initiation of treatment, the patient finally showed some improvement. His leukocytes, ESR and CRP demonstrated a downward trend and were 10.2 × 10^9^/L, 125 mm/hour and 9.1 mg/dL respectively (Table 1). At about the same time, the patient developed swelling and tenderness of his left knee and a knee arthrotomy and washout was performed, the knee aspirate too grew MRSA with similar antibiogram as the previous isolates. Subsequently, fusidic acid was added to vancomycin. Following this, the temperature started to settle. Vancomycin level monitoring was regularly done to evaluate drug toxicity and vancomycin was continued till discharge.

**Table 1:** Acute phase reactants levels during the clinical course of disease

**Time from hospitalization**	**On admission**	**1 week**	**2 week**	**1 month**	**2**^**nd**^**month**	**3 month**	**5 month**	**6 month**	**10 month**	**14 month**
WBC x 10^9^/L	17.7	21.9	18.5	10.2	5.4	6.8	6.9	8.3	8.7	9.1
ESR (mm/h)	>140		>140	125	122	93	34	16	14	25
C-reactive protein (mg/dl)	45.6		19.8	9.1	7.5	2.6		1	1	0.8

Patient was discharged on day 73 of admission with fusidic acid 500mg tds and rifampicin 300 mg tds and was advised non-weight bearing. At 2 weeks post-discharge follow-up, the leukocyte counts, ESR and CRP were 6.8 × 10^9^/L, 93 mm/hour and 2.6 mg/dl respectively. It took 6 months for the ESR and CRP to normalize, indicating active inflammation; requiring 6-months of anti-MRSA therapy. During the subsequent months, the left thigh pain had improved substantially and patient was able to ambulate. Patient was back to work and was able to perform his daily activities without limitations within one year. At 2-year follow-up, he had full function of his left lower limb and there was no evidence of relapse.

### Phenotypic and genotypic characterization of MRSA strain

Antimicrobial susceptibility testing (Oxoid Ltd, Bastingstoke, UK) was performed and interpreted by the Clinical and Laboratory Standards Institute (CLSI) disc diffusion method.

Characterization of SCC*mec* types and detection of *pvl* gene was performed using conditions as previously described [6]. Detection of 20 other virulent genes*; sea, seb, sec, sed, see, seg, seh, sei, sej* (enterotoxin genes); *eta, etb, etd* (exfoliative toxin genes), *tst* (toxic-shock syndrome toxin gene), *efb* (extracellular fibrinogen binding protein), *fnbA, fnbB* (fibronectin binding protein), *cna* (collagen-adhesin gene), *hlg* (γ-hemolysin genes), *ica* (intracellular-adhesin gene) and *sdr* (putative-adhesin gene) was performed by multiplex PCR using GoTaq DNA Polymerase (Promega Madison Wis, USA) following conditions previously described [6-10]. Typing of staphylococcus protein A (*spa*)*, mec-*associated *dru* and multilocus sequence type (MLST) was performed using established protocols as described by web-based electronic database ( http://www.dru-typing.org/; http://saureus.mlst.net/misc/info.asp; http://www.ridom.de/doc/Ridom_spa_sequencing.pdf), whereas typing of accessory gene regulator (*agr*) group was performed as previously described by Moore and Lindsay [10].

## Results of phenotypic and genotypic characterization

**Figure 5:**
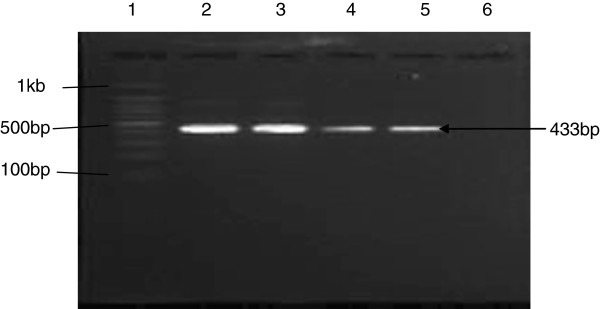
**Agarose gel electrophoresis demonstrating the results of PCR for PVL gene.** Lane 1: 100 bp DNA marker; Lane 2 & 3: positive PVL gene control; Lane 4 & 5: patient’s sample carrying PVL gene; Lane 6: negative PVL gene control.

The isolate was sensitive to non-beta-lactam antibiotics including vancomycin, teicoplanin, rifampicin, fusidic acid, trimethoprim- sulfamethoxazole (TMP-SMX), tetracycline, ciprofloxacin, gentamicin, netilmicin and clindamycin but resistant to erythromycin. Based on the D-zone test, this isolate showed inducible-clindamycin resistance. Molecular characterization of this isolate showed that it belonged to MLST type ST30 and exhibited the SCC*mec* type IV, *spa* type t019, *agr* type III and *dru* type dt10m. This strain, tested positive for 4 virulence genes; *pvl* (cytotoxin) (Figure 5), *sei* (enterotoxin), *fnb*A (fibronectin binding protein) and *hlg* (γ-hemolysin). This strain lacked the exfoliative gene, toxic-shock syndrome toxin gene and the other virulent genes tested.

## Discussion

This previously healthy adult developed an invasive CA-MRSA infection manifesting as bacteraemia, osteomyelitis of femur, pyomyositis and septic arthritis. Osteomyelitis is a rare entity of CA-MRSA presentation, especially so amongst adults. A database search of MEDLINE and Scopus for all years, revealed only 8 cases of acute osteomyelitis caused by CA-MRSA in adults (≥ 18 years). The clinical features of the 9 cases (including the one described here) are shown in Table 2[11-16]. Long bones were most commonly involved (6 femur and 1 humerus). In 2 other patients the osteomyelitis involved the rib and lumbar spine respectively. Pyomyositis was an accompanying manifestation in 5 cases and septic arthritis in 2 patients. Amongst the 9 patients, there was concomitant bacteraemia in 6 patients. In a study involving *Staphylococcus aureus* bacteraemic adults in Taiwan, 16.7% (5/30) of CA-MRSA bacteraemic patients and 20.5% (38/185) of CA-MSSA bacteraemic patients presented with osteomyelitis or septic arthritis [17]. The lack of underlying disease concurred with the experience at most areas where CA-MRSA is described [11-13,15]. Hepatitis C was the only underlying disease described, present in 3 out of 9 patients who also had a history of intravenous drug abuse. The probable source of infection was skin and soft tissue (furunculosis and cellulitis); noted in 6 out of the 9 patients. Risk factors that have been previously identified for CA-MRSA infection includes neonates, school children, university students, athletes, military personnel, cystic fibrosis patients, jail inmates, men who have sex with men (MSM), household contacts, urban undeserved communities, indigenous population and HIV patients [1,4]. MRSA is now increasingly recognized in the animal population and has emerged as possible reservoirs for human MRSA infection in the community, reported in diverse species of animals including domestic pets, livestock, wild birds and other animals [1]. Since no identifiable risk factors were present in our patient, we speculate that he acquired CA-MRSA through extensive direct contact with deer and also possibly through consumption of contaminated deer meat. Although there have not been reported cases of CA-MRSA in deer, other antibiotic resistant bacteria e.g. resistant *E. coli* have been found in wild animals including deer that have not been directly exposed to antibiotics [18]. Our patient was infected with CA-MRSA ST30 which has been found to be associated with pigs [19]. Animals such as deer may constitute an unrecognized source of CA-MRSA infection in natural environment, and more research is needed to assess the importance of deer as reservoirs of MRSA.

**Table 2:** Review of acute osteomyelitis cases caused by CA-MRSA in adults

**No**	**Ref**	**Age/ sex**	**Underlying disease**	**Probable source of entry**	**Symptoms**	**Manifestation**	**Culture source**	**PVL**	**Treatment**	**Outcome**
1	11*	27/M	None	History of furunculosis Contact with gym facilities	Leg pain and fever	Femur osteomyelitis	Bone	+	Vancomycin −8 weeks Surgical debridement	Recovered and no relapse during follow-up
2	11 *	37/M	Hepatitis C Hypertension	History of furunculosis Contact with children with ‘skin bumps’	Leg pain and fever	Femur osteomyelitis	Bone	+	Vancomycin- 8 weeks Surgical debridement	Recovered and no relapse during follow-up
				IVDU						
3	12	45/M	None	NA	Right hemithorax pain and swelling Fever	First rib osteomyelitis, pyomyositis, thoracic wall abscesses, septicaemia	Bone, blood, pus and tissue	+	Clindamycin, tobramycin+ linezolide, vancomycin (given at different times for 6 weeks)	Recovered
									Surgical debridements Abscess drainage	
4	13 *	34/M	None	Axillary furuncle	Thigh pain	Femur osteomyelitis, bacteraemia	Bone and blood	NA	Vancomycin +rifampicin	Recovered and no relapse during follow-up
									– 12 weeks	
									Surgical debridement Intramedullary rod	
5	14 *	46/F	Hypertension	History of furunculosis in patient and daughter	Arm pain	Humerus osteomyelitis with pathological fracture	Bone	NA	Vancomycin+ rifampicin (various combination total of 16 weeks)	Recovered and no relapse during follow-up
									Surgical debridement	
									ORIF	
6	14	28/F	Hepatitis C	History of furunculosis Subcutaneous heroin injection	Thigh pain and swelling	Femur osteomyelitis, pyomyositis, and bacteraemia	Bone and blood	NA	Vancomycin, linezolide, daptomycin (various combination)	Recovered and no relapse during follow-up
									ORIF	
									Surgical debridement	
7	15	22/M	None	Elbow cellulitis Works at military base	Knee and thigh pain Fever	Femur osteomyelitis. septic arthritis of knee bacteraemia, thigh abscess, endocarditis, septic lung emboli, deep vein thrombosis	Wound and blood	+	Vancomycin + clindamycin,	Survived with disabilities
									Vancomycin + linezolid (various combination- total of 8 weeks)	
									Incision and drainage	
8	16	46/M	Hepatitis C with cirrhosis	IVDU	Back pain and fever	Lumbar osteomyelitis, pyomyositis of paraspinal and psoas muscle, spinal epidural abscess, bacteraemia	Blood and pus	NA	Abscess drainage	Recovered and no relapse during follow-up
									Surgical debridement Vancomycin- 6 weeks	
9	Present*	28/M	None	Extensive direct contact with deer	Thigh pain and swelling Fever	Femur osteomyelitis, septic arthritis of knee pyomyositis bacteraemia	Bone, blood, pus and tissue	+	Abscess drainage Arthrotomy	Recovered and no relapse during follow-up
	Case									
									Surgical debridement Vancomycin+fusidic Fusidic + rifampicin- 6 months	
				Consumed deer meet						

*Suspected primary bone malignancy.

NA- not available.

One noteworthy finding is that CA-MRSA osteomyelitis involving the long bones has a propensity to mimic malignant bone tumors radiologically (5 out of the 7 long bone osteomyelitis in our review). Symptoms such as fever, bone pain, loss of weight, loss of appetite are non-specific and may not differentiate osteomyelitis or bone tumors. Pain of moderate to severe intensity was a constant feature in all patients (Table 2) and may suggest the diagnosis of osteomyelitis. Our patient was initially suspected to have osteosarcoma based on radiological findings, which was ruled out by a needle biopsy. Biopsy also identified the causative pathogen so that appropriate antibiotic treatment could be administered. Vancomycin is the first-line intravenous drug for severe CA-MRSA infections [1]. Oral agents recommended for treatment of CA-MRSA infections include clindamycin, doxycycline, co-trimoxazole, rifampicin and fusidic acid [2]. Rifampicin or fusidic acid should not be used as monotherapy because of the possible emergence of resistance [2]. Clindamycin should be used with caution since erythromycin resistant isolates can have inducible resistance to clindamycin [1]. This was the case in our patient as determined by the D-zone test.

Our patient had an uneventful recovery with no evidence of relapse at 2 year follow-up. Peyrani et al. [20] conducted a study involving 50 patients with MRSA osteomyelitis of which 54% were due to MRSA USA-300 (mainly CA-MRSA, SCC*mec* type IV). Although the rate of treatment failure was similar for patients with MRSA USA-300 osteomyelitis versus patients with MRSA non–USA-300 (mainly healthcare-associated, SCC*mec* type III), patients with more severe initial clinical presentation were most likely infected with MRSA USA-300 strains. In another study comparing CA-MRSA and CA-MSSA osteomyelitis, there was no difference in the final outcome of patients from these 2 groups, although the subset of CA-MRSA osteomyelitis patients were more likely to have complications such as chronic osteomyelitis and deep vein thrombosis [21].

Testing for *pvl* gene was done on 5 patients and all 5 tested positive (Table 2). PVL is a cytotoxin responsible for the destruction of leucocytes and is encoded by *pvl* genes namely *lukS-PV* and *lukF-PV*[2] and plays an important role in the pathogenesis of severe invasive infections such as skin and soft tissue infections, osteomyelitis and necrotizing pneumonia, contributing to tissue necrosis and abscess formation. In a study involving acute hematogenous *S. aureus* osteomyelitis in children, *pvl* positive isolates had significantly higher ESR and CRP at presentation and were more likely to have a blood culture positive and contiguous pyomyositis or myositis [5]; concluding that *pvl* gene is strongly associated with the severity of acute osteomyelitis. Our patient had very high inflammatory markers at presentation. With appropriate treatment, these markers gradually demonstrated a downward trend. It took 6 months for these markers to almost normalize, necessitating 6 months of anti-MRSA treatment. Early and prolonged administration of anti-MRSA treatment until all inflammatory parameters normalized contributed to a favorable outcome of our patient. The CA-MRSA isolated from our patient was *pvl* positive, ST30/*spa*19 which have been found to be highly virulent and shown to be associated with life-threatening invasive infections, including pelvic abscesses, osteomyelitis and pneumonia in children and adolescent athletes [22].

The MRSA strain of our patient was also tested positive for *sei*, *fnbA* and *hlg*. Fibronectin-binding proteins (FnBPA and FnBPB) encoded by *fbp*A and *fbp*B genes play a role in enhanced bacterial adherence to fibronectin and invasion of host cells including epithelial cells and fibroblasts and were significantly more frequently detected in *S. aureus* invasive strains (responsible for osteomyelitis, septic arthritis and endocarditis) compared with nasal carriage isolates [5,8]. γ- hemolysin are cytolysins also with both leukocidin and hemolysin properties [4]. In addition to being classified as members of the pyrogenic toxin superantigen family, Staphylococcal enterotoxins (SEs) are emetic toxins and *se* genes are frequently detected in isolates that originated from food poisoning outbreaks. Although the *sei* gene was detected, our patient did not have manifestations of food-poisoning as the *sei*- harboring isolate may not have produced enough SEI enterotoxins to cause food poisoning [9].

## Conclusion

This case highlights the importance of a high index of suspicion for early diagnosis of CA-MRSA, especially in the context of an immunocompetent adult. Clinicians need to be attentive that beyond the most commonly encountered skin and soft tissue infections, *pvl* positive CA-MRSA can lead to invasive life-threatening disease. Heightened vigilance is needed for CA-MRSA osteomyelitis of long bones in adults as it is not uncommon for this disease to mimic primary bone malignancy, which can be ruled out based on bone biopsy supported by microbiological evidence. Cure is achievable with early appropriate antibiotics guided by inflammatory markers.

### Consent

Written informed consent was obtained from the patient for publication of this Case report and accompanying images. A copy of the written consent is available for review by the Series Editor of this journal.

## Competing interests

The authors declare that they have no competing interests.

## Authors’ contributions

AD conceived the idea, wrote and edited the final manuscript. VAS and AM were the Orthopaedic Surgeons who treated and planned the management of the patient and were involved in critical appraisal of the manuscript. MYY coordinated sample collection from the wards, supervised all the laboratory diagnosis and helped draft the manuscript. KTL performed the phenotypic and genotypic characterization of MRSA strain and helped draft the manuscript. KLT supervised the phenotypic and genotypic characterization of MRSA strain and was involved in drafting the manuscript. All authors read and approved the final manuscript.

## Funding

No funding source.

## Pre-publication history

The pre-publication history for this paper can be accessed here:

http://www.biomedcentral.com/1471-2334/12/270/prepub
